# Development of a Novel Quantitative Structure-Activity Relationship Model to Accurately Predict Pulmonary Absorption and Replace Routine Use of the Isolated Perfused Respiring Rat Lung Model

**DOI:** 10.1007/s11095-016-1983-4

**Published:** 2016-07-11

**Authors:** Chris D. Edwards, Chris Luscombe, Peter Eddershaw, Edith M. Hessel

**Affiliations:** 1 Refractory Respiratory Inflammation DPU, GlaxoSmithKline Medicines Research Centre, Gunnels Wood Road, Stevenage, Hertfordshire SG1 2NY UK; 2 Chemical Sciences, GlaxoSmithKline, Stevenage, UK

**Keywords:** intratracheal delivery, isolated perfused lung, physicochemical properties, pulmonary absorption, quantitative structure-activity relationship model

## Abstract

**Purpose:**

We developed and tested a novel Quantitative Structure-Activity Relationship (QSAR) model to better understand the physicochemical drivers of pulmonary absorption, and to facilitate compound design through improved prediction of absorption. The model was tested using a large array of both existing and newly designed compounds.

**Methods:**

Pulmonary absorption data was generated using the isolated perfused respiring rat lung (IPRLu) model for 82 drug discovery compounds and 17 marketed drugs. This dataset was used to build a novel QSAR model based on calculated physicochemical properties. A further 9 compounds were used to test the model’s predictive capability.

**Results:**

The QSAR model performed well on the 9 compounds in the “Test set” with a predicted *versus* observed correlation of R^2^ = 0.85, and >65% of compounds correctly categorised. Calculated descriptors associated with permeability and hydrophobicity positively correlated with pulmonary absorption, whereas those associated with charge, ionisation and size negatively correlated.

**Conclusions:**

The novel QSAR model described here can replace routine generation of IPRLu model data for ranking and classifying compounds prior to synthesis. It will also provide scientists working in the field of inhaled drug discovery with a deeper understanding of the physicochemical drivers of pulmonary absorption based on a relevant respiratory compound dataset.

## Introduction

Reducing compound attrition is a highly desirable goal in drug discovery. This aim has been facilitated by the emergence of physicochemical property-based guidelines such as the Lipinski Rule of 5 (1), simple ADMET rules of thumb (2–4) and rules for reducing receptor promiscuity (5–7). Hughes *et al*. (5) showed for example that targeting lower lipophilicity (cLogP <3) and greater polarity (polar surface area >75) could help reduce toxicity-based attrition. The physicochemical properties of medicines delivered *via* the oral route are generally well understood because this area has been the focus of numerous analyses on preclinical and clinical datasets (8–11).

Conversely, there is relatively little information in the literature on the optimal physicochemical properties of inhaled drugs. It can be highly advantageous for certain medicines to specifically target an organ rather than the systemic compartment, for example in respiratory diseases where the disease is occurring in the lung. There are also several examples, extensively reviewed by Patton and Byron (12,13), where pulmonary delivery has been selected as a way of delivering systemically acting drugs. Further literature concerning the inhaled route of administration mostly describes non-drug like molecules, and is therefore not very informative for small molecule drug discovery (14–17).

To aid design of small molecule inhaled drugs, a similar set of guidelines to those described for oral drugs would be advantageous. Colleagues at GSK have published a physicochemical analysis of 29 inhaled/intranasal and 52 oral marketed respiratory drugs (18), concluding that compounds administered *via* the inhaled/intranasal routes have a higher polar surface area, a higher molecular weight, and a trend towards lower lipophilicity, when compared with their orally administered counterparts. Of the 29 inhaled/intranasal drugs, 69% were either glucocorticoids, β2 agonists, or muscarinic antagonists, reflecting the dominance of these medicines as current standard of care for respiratory diseases.

The isolated perfused rat lung (IPRLu) model is an *ex vivo* tool for investigating rate and extent of compound absorption through the lung following intratracheal instillation in rats. It has been extensively used to compare lung retention and absorption rates between compounds (19–23). Ventilation of the lungs ensures physiological relevance whilst the intact vasculature of the IPRLu model allows evaluation of the fate of drugs without the influence of extra-pulmonary factors such as hepatic clearance.

Studies comparing the IPRLu model with *in vivo* models report good consistency across the data and suggest the IPRLu model is a suitable tool for investigating lung absorption. Tronde (24) reported that the absorption half-life obtained with the IPRLu model correlated with the observed *in vivo* lung absorption half-life based on a total of 5 compounds in rats. Comparable data have also been reported between an IPRLu model and an *in vivo* model following administration of fluticasone furoate as a dry powder (25).

Tronde (24) also published a Partial Least Squares (PLS) model predicting absorption rate in the IPRLu model from measured LogP_app_, and the descriptors %PSA and cLogD_7.4_. This study however, was based on a limited dataset of 10 compounds of which only 3 were inhaled drugs.

Given the current limited characterization of inhaled drugs in physicochemical space, we sought to use data generated in our laboratory with the IPRLu model to produce a computational model able to predict pulmonary absorption, thus obviating the need to use *ex vivo* or *in vivo* models. This in silico analysis would improve characterisation of existing inhaled drugs and facilitate the design of novel drugs.

Here we present a novel in silico model that we constructed using the largest and most relevant data set available so far, comprising both marketed inhaled drugs with well known mechanisms of action, and novel inhaled compounds with thus far less established mechanisms of action. We subsequently validated our in silico model using a compound “Test set”, which generated robust prospective predictions, confirming the applicability of our model.

## Materials and Methods

### Isolated Perfused Rat Lung Model

All animal studies were ethically reviewed and carried out in accordance with the Animals (Scientific Procedures) Act 1986 and the GSK Policy on the Care, Welfare and Treatment of Animals.

Male CD rats (275–300 g) were obtained from Charles River (Margate, Kent, UK) and housed under standard conditions. The IPRLu model, including surgery, dosing and sample collection, was performed by the Laboratory Animal Sciences department at GSK. Methodology was based on that described by Tronde *et al.* (23;24) but with lungs remaining *in situ* and intratracheal instillation as the dosing technique. Prior to surgery male CD rats were anaesthetised with intraperitoneal ketamine and medetomidine (Dormitor^TM^) at 75 ml/kg and 0.5 ml/kg respectively. In addition, 50U of heparin was administered intravenously *via* a tail vein. Following confirmation of anaesthesia, the front of the rib cage was removed to access heart and lungs, and the trachea cannulated and secured with a ligature. Respiration was transferred to a positive pressure small animal ventilator (Harvard Apparatus Ltd, Edenbridge, Kent, UK) and maintained at 40 breaths per minute with a tidal volume of 1.8 ml. Following exsanguination, the pulmonary artery and vein were cannulated through incisions in the right and left ventricles in order to isolate the blood pulmonary circulatory system. The rat was then raised and maintained at an angle of 45° using a custom made elevating chamber similar to that described by Brain *et al*. (26). Perfusion of the isolated lung vasculature was performed with Krebs-Ringer bicarbonate buffer (3% bovine serum albumin (BSA), pH7.4, 37°C), pumped at 10 ml/min (27) using a Harvard rodent blood pump (Model 1407, Harvard Apparatus, Holliston, MA, USA). After an initial first pass to flush blood from the lung vasculature, the perfusion buffer was then recirculated for approximately 10 minutes to equilibrate and attain the required flow rate. Lungs were visually inspected for leaks and signs of oedema (27). Compounds were administered either discretely or co-formulated (2–4 compounds) and experiments were carried out in duplicate. Doses (100 μL) were administered to the trachea slightly above the bifurcation point by introduction of a 50 mm needle Hamilton syringe into the tracheal cannula followed by 3 x 1 ml of air. The tracheal cannula was then reattached to the ventilator and total perfusate was collected over 1 min intervals for 20 mins. Perfusate aliquots (1 ml) were frozen (-20°C) along with terminal lung samples prior to analysis.

Homogenised lung and perfusate samples underwent protein precipitation by addition of a 3-fold excess of acetonitrile containing an internal standard. Following either filtration or centrifugation the resulting filtrate or supernatant was typically dried down under a nitrogen stream and then reconstituted in the mobile phase used for separation by HPLC. Following sample preparation lung and perfusate samples, along with residual dose formulations (pre- and post-centrifugation) were analysed by HPLC tandem mass spectrometry (LC-MS/MS), typically using a generic reverse phase chromatography gradient method with separation on a C18 column (50 x 2.1 mm, 3 μm) and an acidified mobile phase.

### Compounds

All 108 compounds used in our experiments were either from inhaled respiratory drug discovery programmes (91) or marketed drugs (17), synthesized or purchased by GlaxoSmithKline and dosed between 7 and 40 μg. The “Training set” contained 98 compounds of which 82 were discovery compounds and 16 were marketed compounds. The “Test set” consisted of 9 discovery compounds. Of the 17 marketed compounds all were included in the “Training set” with the exception of Tiotropium for which solubility had not been measured, therefore a value for % solubilised dose in perfusate (%SDiP) was not available.

All compounds were formulated in an aqueous vehicle, the majority (79%) containing 0.2% Tween 80 in either water or saline. Due to the diverse nature of the molecules investigated, formulations were a mix of solutions and suspensions ranging from <1 to 100% compound in solution. Compound solubility effects were investigated using a tool compound available in three micronised salt forms with varying solubilities. The free base (FB), hydroxynapthoate (HNA) and hydrochloride (HCl) forms were each administered to the IPRLu model at a dose equivalent to 15 μg free base.

### Data Handling

The cumulative amount of parent compound crossing the lung into the perfusate by the end of the 20 minute experiment was expressed as % of the total dose or as % of the dose originally in solution (solubilised dose). The relative rate of absorption across the lungs was also calculated, the percentage of the administered dose remaining in the lungs was plotted semilogarithimically against time and the initial rate of absorption expressed as a half-life.

### Development of QSAR Model

To determine the main drivers of absorption of compounds in the IPRLu model, multivariate analysis was carried out on the IPRLu model output together with calculated physicochemical parameters. The statistical method used to build the QSAR model was an orthogonal partial least squares (OPLS) regression based modelling approach (28,29) using SIMCA-P+ software (Umetrics, Umeå, Sweden).

The molecular structure of each compound in the dataset was used to calculate 39 two-dimensional physicochemical descriptors that generally describe lipophilicity, hydrogen-bonding, size, shape, charge and atom composition, and included descriptors calculated using ACD/Labs software (Toronto, Ontario, Canada) and Abraham’s molecular descriptors (30). These properties were then combined with a set of in silico ADME related endpoints (gastro-intestinal absorption, Lipinski’s Rule of 5 (1), extent of protein or tissue binding, substrate for active efflux transporter P-glycoprotein, volume of distribution, solubility and permeability) to form the x-block variables used for modelling purposes.

Data from the 98 compounds in the “Training set” consisted of a total of 107 observations of which 9 were repeat compounds with data generated across the IPRLu model timeframe, their inclusion helped assess variability in the model. Using this data OPLS models were generated using the x block variables to model the log transformed y variables (*i.e.* model output): Log% solubilised dose in perfusate at 20 mins (Log%SDiP), Log% total dose in perfusate at 20 mins (Log%TDiP), and lung absorption half-life. A model predicting Log%SDiP was taken forward and from the original set of 49 x variables, a single round of feature selection was carried out based on the variable importance plot from the initial OPLS model to generate a more parsimonious model comprising of 20 descriptors and 6 ADME model outputs. The number of components was determined automatically within SIMCA-P+ using a “leave many out” cross validation procedure to assess their individual significance. To test the robustness of the model, a randomisation test was performed 20 times using the “Validate” function within SIMCA-P+ on the y dataset.

A further 9 compounds for which IPRLu model data was generated (*i.e.* not included in the “Training set”) were used as a “Test set” to assess the predictivity of the QSAR model.

## Results

### Diversity in Pulmonary Absorption Across 17 Marketed Compounds Using the IPRLu Model

The rate and extent of lung absorption of 17 marketed drugs along with 82 discovery compounds were measured in the IPRLu model and used to build an in silico model to predict pulmonary absorption.

Data generated with the IPRLu model on 17 marketed drugs (14 out of 17 designed for inhaled delivery) are shown in Table I. All the compounds display a moderate to high extent of total dose in the perfusate at 20 minutes (10–100%), in keeping with the in-house observation that compounds displaying < <10% carry a high risk of accumulation in the lung upon repeat dosing, which can lead to developability issues and high attrition rates. The marketed compound set can be classified into two groups according to their solubility. The first group are reasonably soluble in the dose formulation (>80% of the parent compound in solution, determined by dose analysis pre- and post-centrifugation) and display similar values for %SDiP and %TDiP. The second group are only partially soluble in the dose formulation (<80% of the parent compound in solution) and therefore display greater values for %SDiP compared to %TDiP.

**Table I:** Data for Marketed Drugs Generated in the IPRLu Model and Included in the OPLS Model “Training set”, Mean (*n* = 2) Data are Displayed with the Range Quoted in Brackets

Drug	%TDiP	%SDiP	% Dose in solution	Lung T1/2 (mins)	Recovery of total dose (%)^a^	Calculated LogP^b^
Indacaterol	11 (10,11)	12 (12)	88	273 (255–291)	91 (77–104)	3.03
Ambroxol	38 (14–61)	41 (18–64)	95	39 (14–64)	70 (66–74)	2.65
Formoterol Fumerate	47 (42–52)	47 (42–52)	100	24 (22–26)	73 (70–76)	0.83
Ipratropium Bromide	49 (34–64)	50^c^	100	23 (14–32)		–1.82
Tiotropium Bromide^d^	61 (59–62)			16 (15,16)		–1.76
Amiloride	62 (61–62)	62 (61–62)	100	8 (7–10)	81 (76–85)	-0.5
Lidocaine^e^	75 (64–82)	75^c^	100	4 (4,5)		1.54
Zanamivir^f^	100 (100–100)	100	100	7 (5–8)	104 (100–107)	–6.54
Flunisolide	41 (28–53)	96 (66–126)	42	15 (7–22)	139 (135–142)	1.56
Montelukast	67 (57–78)	92 (78–106)	73	42 (23–60)	77 (70–85)	8.49
Fluticasone Propionate	10 (10)	2000^c^	0.5	115 (98–131)		3.72
Fluticasone Furoate	10 (7–12)	300^c^	3.2	153 (112–194)		4.13
Nedocromil	32 (23–40)	125 (83–166)	24	32 (24–40)	85 (79–90)	2.5
Tacrolimus (prograf)	38 (38)	178 (176–180)	21	28 (27,28)		5.59
Budesonide	77 (76–79)	551 (540–561)	14	3.3 (3,4)	84 (82–87)	2.73
Salmeterol	15 (6–24)	41 (16–66)	36	146 (59–233)	69 (61–76)	3.59
Salbutamol	42 (33–51)	77 (60–93)	55	21 (13–28)	78 (75–81)	0.32

^a^Data only available for compounds where lung analysis was carried out to confirm recovery

^b^Calculated using Chemaxon v5.4.1.1 (http://www.chemaxon.com/)

^c^Solubility in dose vehicle was determined post study on a discrete weighing of the compound

^d^Solubility of Tiotropium was not measured in the IPRLu model experiment, therefore it was not included in the “Training set”. Safety data sheet quotes solubility to be ~5 mg/ml in PBS (pH 7.2) https://www.caymanchem.com/msdss/15773m.pdf

^e^Lidocaine data is from n = 4

^f^Zanamivir dose analysis failed, expected to be fully solubilised in the dose vehicle (250 μg/ml 0.2% Tween 80 in saline) due to its zwitterionic nature. Merck Index states solubility of zanamivir to be 18 mg/ml in water

The marketed compounds could also be differentiated based on the mean lipophiciity of the two groups. LogP was calculated using Chemaxon v5.4.1.1 (http://www.chemaxon.com/) and the difference between the 2 groups was deemed significant using a non-paired *t*-test assuming equal variance. For the first group, where the dose is mainly in solution (*i.e.* %SDiP ≅ %TDiP) the compounds display a mean LogP value of -0.3 and include: indacaterol, ambroxol, formoterol fumerate, ipratropium bromide, amiloride, lidocaine and zanamivir. The second group (%SDiP > %TDiP) of compounds display a mean LogP value of 3.6 and include: flunisolide, montelukast, fluticasone propionate, fluticasone furoate, nedocromil, tacrolimus, budesonide, salmeterol and salbutamol. Tiotropium bromide was not included in this analysis as a %SDiP was not available.

### Importance of solubility as a driver of pulmonary absorption

**Fig. 1:**
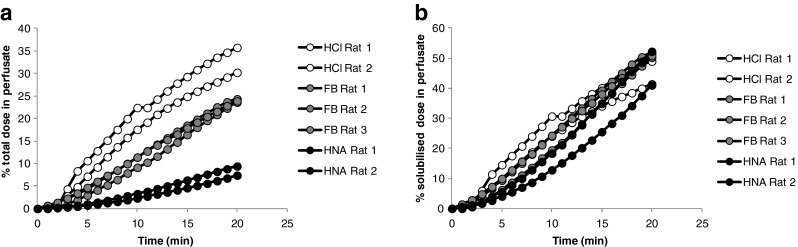
IPRLu model profiles for different salt forms of the same parent drug discovery compound expressed as (**a**) % dose in perfusate, where a 6 fold difference is noted between salt forms at 20 mins and (**b**) %SDiP *i.e.* normalised for the amount of dose in solution, where profiles are comparable. HNA = hydroxynapthoate, FB = free base, HCl = hydrochloride salt.

The importance of solubility as a key determinant of pulmonary absorption was highlighted with a tool discovery compound which displayed a range of IPRLu model profiles driven by differences in solubility of the specific micronised salt form administered (Fig. 1). Pulmonary absorption, expressed as %TDiP, correlated with the extent of parent compound in solution in the dose vehicle: 18, 47 and 73% for the HNA, FB and HCl salt forms respectively. Furthermore when the IPRLu model data were normalized to the amount of the administered dose in solution (%SDiP), the parent compound profiles were similar regardless of the salt form administered, confirming that similar proportions of the parent compound in solution crossed the lungs into the perfusate.

### Development of QSAR model to predict pulmonary absorption

By applying multivariate analysis on a “Training set” of 98 compounds we have identified a comprehensive series of physicochemical descriptors that correlate with pulmonary absorption in the IPRLu model.

The “Training set” comprised of a physicochemically diverse set of 98 compounds consisting of 7 zwitterions, 8 acids, 31 bases and 52 neutral compounds, with cLogP values ranging from -3.7 to 9.1 and cLogDpH7.4 values ranging from -4.4 to 6.5. Total polar surface area (tPSA) values ranged from 24 to 198 and molecular weight values ranged from 177 to 842 (see Table II).

**Table II:** Range of Physicochemical Properties and IPRLu Model Endpoints Across the 98 Compounds (7 Zwitterions, 8 Acids, 31 Bases and 52 Neutral) in the “Training Set”

Physicochemical property	IPRLu model endpoint	Mean	Lower range	Upper range
cLogP		3.2	–3.7	9.1
cLogD pH7.4		2.1	–4.4	6.5
M.Wt.		507	177	842
tPSA		105	24	198
	%TDiP	32	0.1	100
	%SDiP	164	0.1	2400
	Lung absorption half-life (mins)	212	3.3	5210

Ionisation classes were predicted and cLogP/cLogD values calculated using the ACDlabs software ACD_v11: Advanced Chemistry Development, Inc. 8 King Street East, Suite 107, Toronto, Ontario, Canada M5C 1B5. tPSA = total Polar Surface Area

The diverse nature of the compound set resulted in a broad range of values for each of the IPRLu model endpoints with %TDiP values ranging from 0.1 to 100, %SDiP values ranging from 0.1 to 2400 and lung absorption half-life values ranging from 3.3 to 5210 minutes (see Table II). Initially, QSAR models were generated on the log transformed data for each of the three endpoint parameters from the IPRLu model. We then selected the best performing QSAR model based on the R^2^ value, which was an OPLS model predicting Log%SDiP. Having already established the importance of solubility as a driver of pulmonary absorption, we then focused on the extent of solubilised dose in perfusate which enabled investigation into the inherent drivers of pulmonary permeability.

**Fig. 2:**
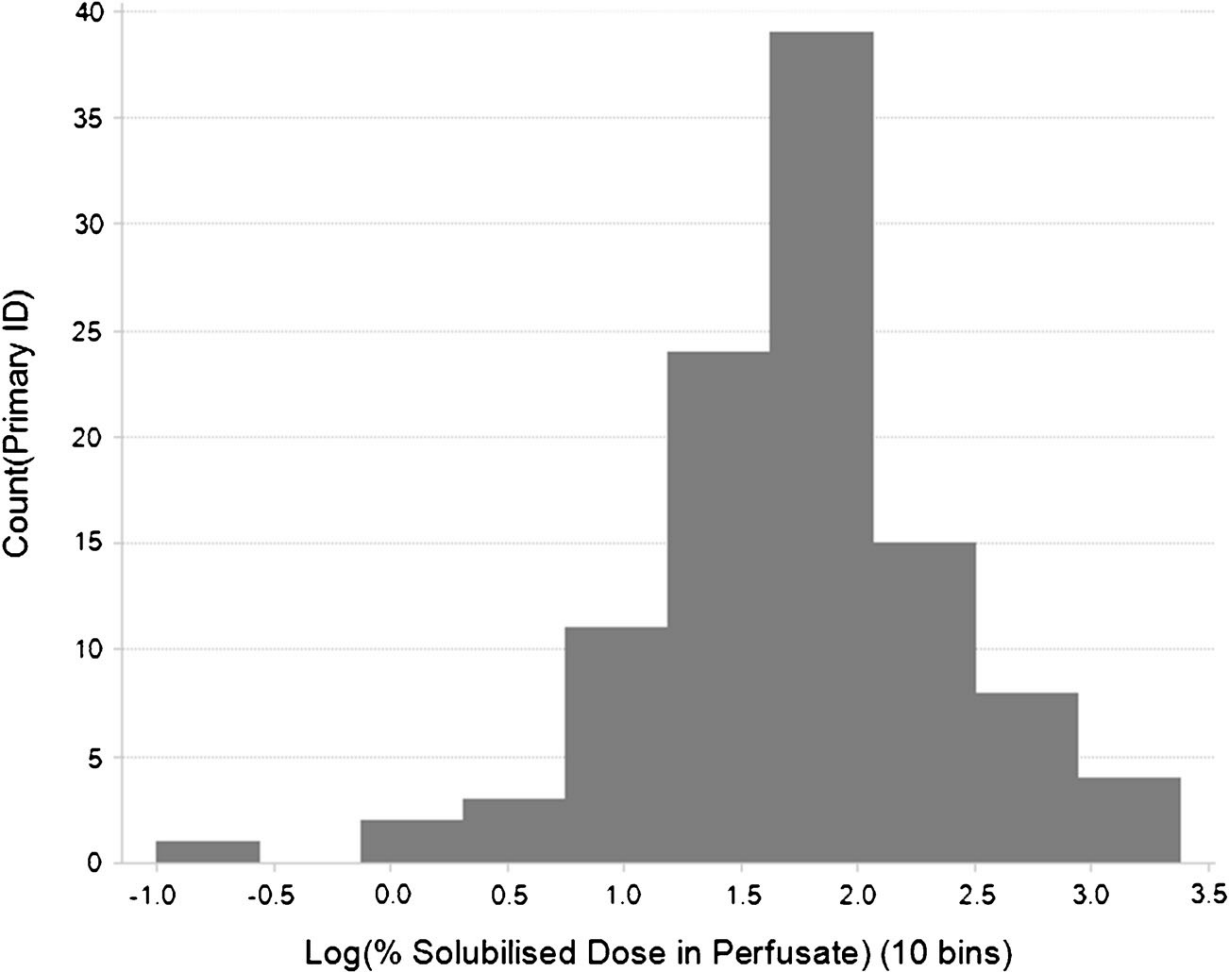
Distribution of data in training dataset “Log%SDiP”.

**Fig. 3:**
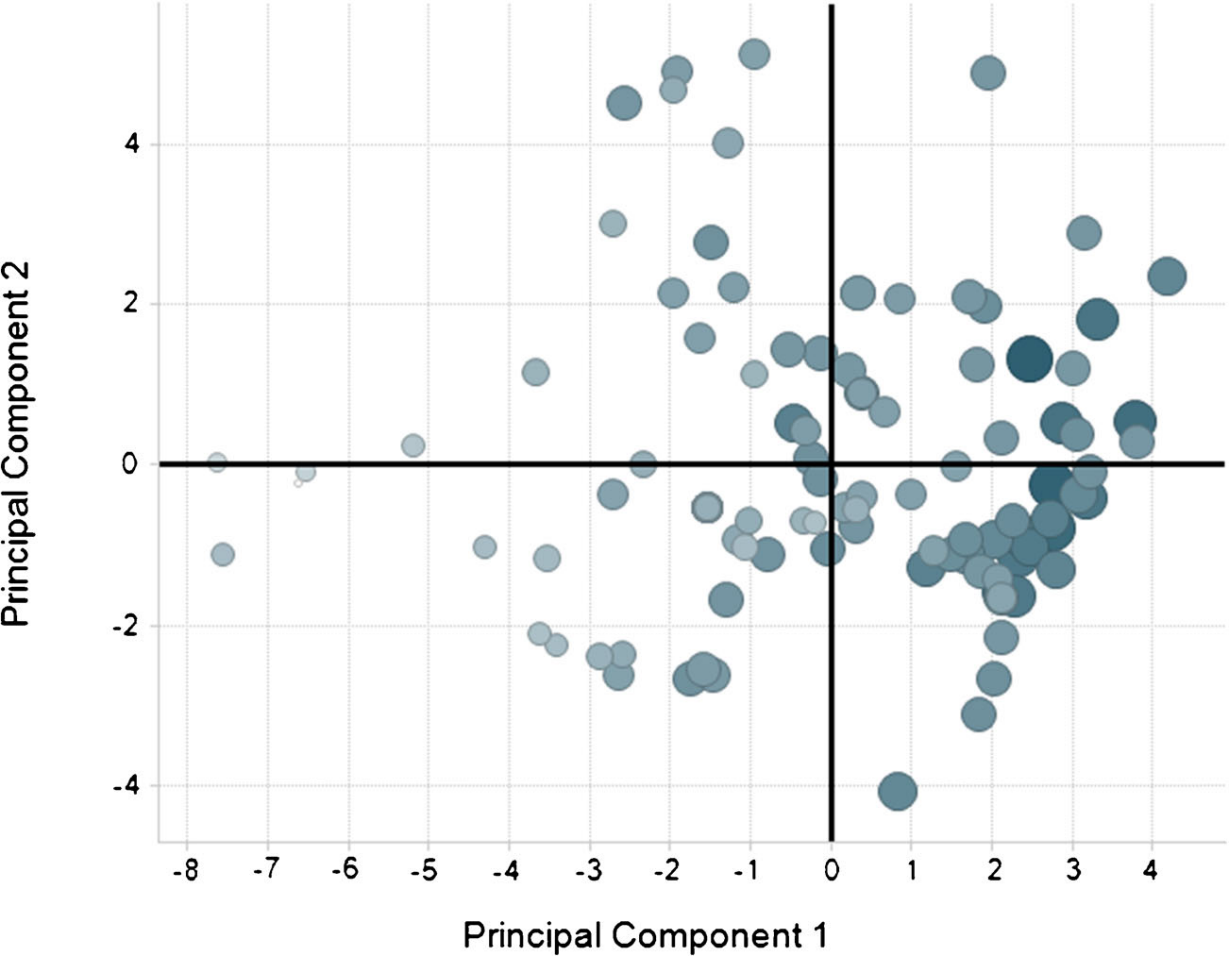
The scores plot from the resulting 2 component OPLS model generated within SIMCA-P+ on the 98 compounds where increasing size and increasing blue intensity of the spots are equated with the size of the response Log%SDiP.

**Fig. 4:**
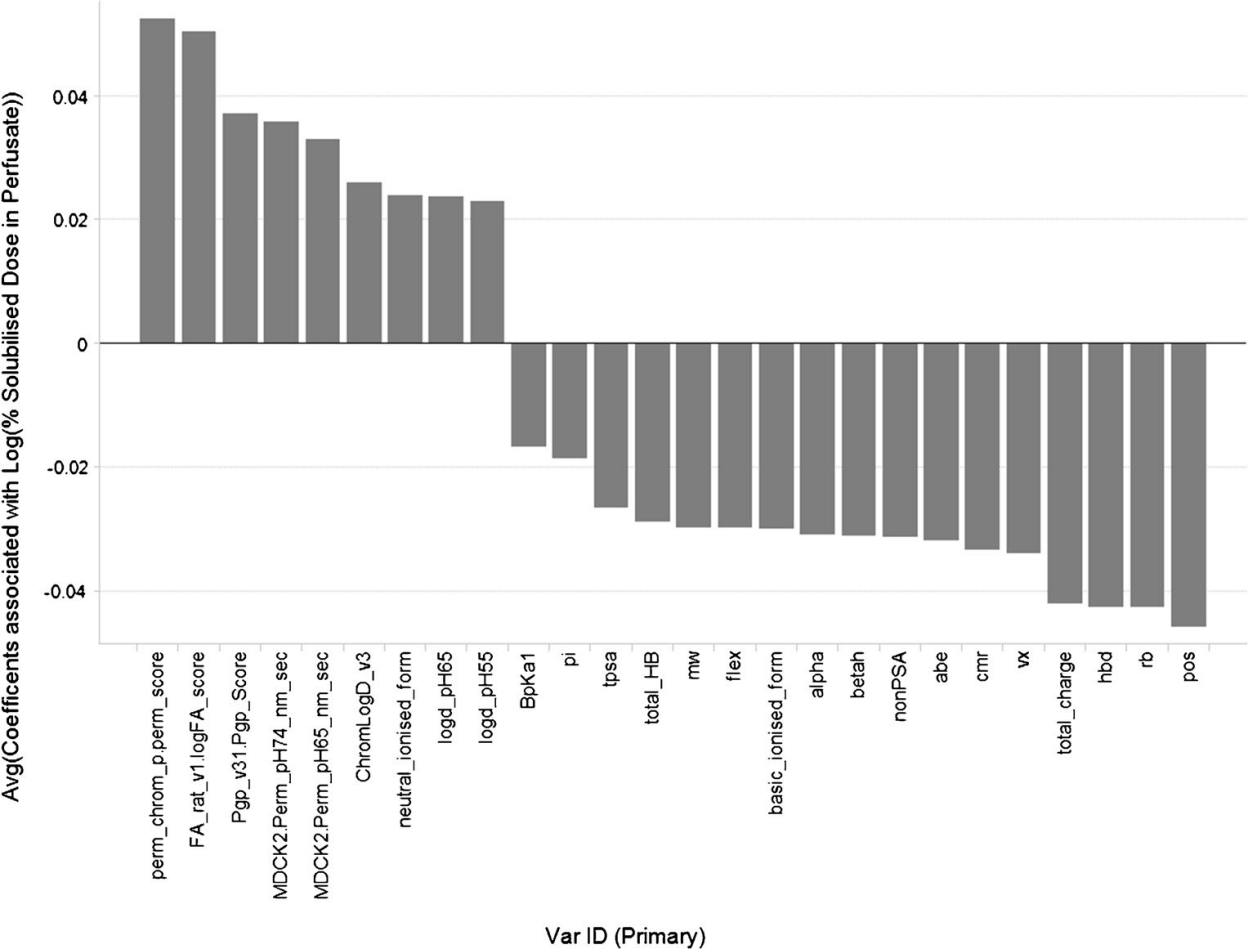
OPLS model coefficient plot displaying the contribution of each descriptor to the model components and whether each descriptor correlates positively or negatively with Log%SDiP.

The distribution of the 98 compound “Training set” is shown in Fig. 2 and reflects a normal distribution of Log%SDiP data. The associated scores plot of the resulting OPLS model is displayed in Fig. 3. The scores plot displays the relationships between different compounds; the x variables in this model are condensed into the 2 principal components plotted and describe the physicochemical space. Adjacent compounds in the scores plot have similar physicochemical properties. A consequence of using OPLS is that all the attributed variability in the y data variable Log%SDiP is described by principal component 1. As a result this leads to the differentiation in the size of the response correlating from left to right along principal component 1. The contribution of each of the 20 descriptors and 6 ADME model outputs of the x variable block are shown in the regression coefficients plot in Fig. 4. Descriptors that positively correlate with Log%SDiP include: extent of absorption in the rat following oral administration (FA_rat_v1.logFA_score), permeability (perm_chrom_p.perm_score, MDCK2.Perm_pH74_nm_sec, MDCK2.Perm_pH64_nm_sec), substrates of the active efflux transporter Pgp (Pgp_v31.Pgp_Score), hydrophobicity (Chrom LogD_v3.value, logd_pH55_acd, logd_pH65_acd), and extent of compound residing in the neutral or unionised form (neutral_ionised_form). These positive correlating descriptors and model outputs are mainly associated with permeability and hydrophobicity. Descriptors that negatively correlate with Log%SDiP include: Bpka1, basic_ionised_form, abe, cmr, mw, tpsa, hbd, pos, rb, flex, alpha, betah, pi, vx, total_HB, total_charge and nonPSA. These negative correlating descriptors are mainly associated with charge, ionisation and size. More details of the different in-house QSAR models and descriptors used to build this OPLS model are shown in Table III.

**Table III:** Information Around the in-House QSAR Models and Descriptors used to Build the Log%SDiP OPLS Model

QSAR model / Descriptor	Details	Coefficients with regards to IPRLu QSAR model
FA_rat_v1.logFA_score	Output from an inhouse QSAR model that predicts fraction absorbed in the rat, this model was built on a GSK rat oral bioavailability dataset with total clearance values of less than 10 ml/min/kg and therefore minimal first pass effect.	+
Perm_chrom_p.perm_score	An inhouse PLS-Discriminant analysis model which predicts permeability and oral absorption of compounds.	+
MDCK2.Perm_pH74_nm_sec MDCK2.Perm_pH64_nm_sec	Inhouse QSAR models which predict passive permeability across a madine darby canine kidney (MDCK) cell monolayer (31) with donor solutions buffered at pH7.4 and pH6.4.	+
Pgp_v31.Pgp_Score	An inhouse PLS-Discriminant analysis model which predicts the likelihood of a compound being a substrate for the ABC active efflux transporter P-glycoprotein.	+
Chrom LogD_v3.value	An inhouse OPLS QSAR model that predicts hydrophobicity, the model was built on a GSK dataset of chromatographic hydrophobicity index (CHI) measurements (32) which are converted to a chromLogD value (4).	+
neutral_ionised_form	% of the molecule in the neutral form at pH7.4. pKa’s calculated using the ACDlabs software^a^ ACD_v11.	+
logd_pH55_acd	Calculated logD value from the ACDlabs software at pH5.5	+
logd_pH65_acd	Calculated logD value from the ACDlabs software at pH6.5	+
Bpka1_modified	The most basic pka value calculated using ACDlabs software ACD_v11	–
basic_ionised_form	% of the molecule in the acidic form at pH7.4 pKa’s calculated using the ACDlabs software ACD_v11.	–
abe	Andrews Binding energy (33).	–
cmr	Calculated molar refraction	–
mw	Average molecular weight of parent	–
tpsa	Polar surface area calculated by the method of Ertl (34).	–
hbd	Count of the number of hydrogen bond acceptors in a molecule.	–
pos	The count of the number of positively ionisable/charged groups in a molecule.	–
rb	count of the number of rotatable bonds in a molecule.	–
flex	related to the ratio of the number of rotatable bonds to total bonds = int(100*rotatable_bonds/total_bonds)	-
alpha	An Abraham’s molecular descriptor relating to Hydrogen Bond Acidity (hydrogen bond donors) (30).	-
betah	An Abraham’s molecular descriptor relating to Hydrogen Bond Basicity (hydrogen bond acceptors) (30).	-
pi	An Abraham’s molecular descriptor relating to the dipolarity/dipolarizability (30).	-
vx	An Abraham’s molecular descriptor relating to McGowan characteristic volume (size) (30).	-
total_HB	Sum of hydrogen bond donors and acceptors (physchem_desc.hba + physchem_desc.hbd)	-
total_charge	Sum of positive and negative charges (physchem_desc.neg + physchem_desc.pos)	-
nonPSA	Approximation of total surface area - polar surface area.	-

^a^ACDlabs software: Advanced Chemistry Development, Inc. 8 King Street East, Suite 107, Toronto, Ontario, Canada M5C 1B5

The statistical output from the OPLS model is shown in Table IV and displays a R^2^ of 0.621 and a Q^2^ (*i.e.* predictivity) of 0.491. The authors recognise that these statistics are not representative of a highly predictive regression model, but feel that they are sufficient for a semi-quantitative ranking and classification based predictive assessment. Moreover, the statistics obtained are typical for an ADME model where the output, Log%SDiP, like other ADME endpoints has an inherent variability and multiple factors influencing it.

**Table IV:** Statistical Output from SIMCA for the Final OPLS Predictive Model for Log%SDiP, OPLS Observations (N) = 107, Variables (K) = 27 (X = 26, Y = 1)

A	R2X	R2X(cum)	Eigenvalues	R2Y	R2Y(cum)	Q2	Limit	Q2(cum)	Significance
0	Cent.			Cent.					
1 + 0	0.462	0.462	6.26	0.34	0.34	0.322	0	0.322	R1
Rotation	–0.221	0.241			0.621			0.491	
1 + 1	0.26	0.26	6.77	0.0722	0.0722	0.038	0	0.038	R1
1 + 2	0.143	0.403	3.71	0.119	0.192	0.0719	0	0.11	R1
1 + 3	0.0884	0.492	2.3	0.0412	0.233	0.0403	0	0.15	R1
1 + 4	0.0809	0.572	2.1	0.0328	0.266	0.0117	0	0.162	R1
1 + 5	0.0549	0.627	1.43	0.0153	0.281	0.0074	0	0.169	R1
Sum		0.868			0.621			0.491	

**Fig. 5:**
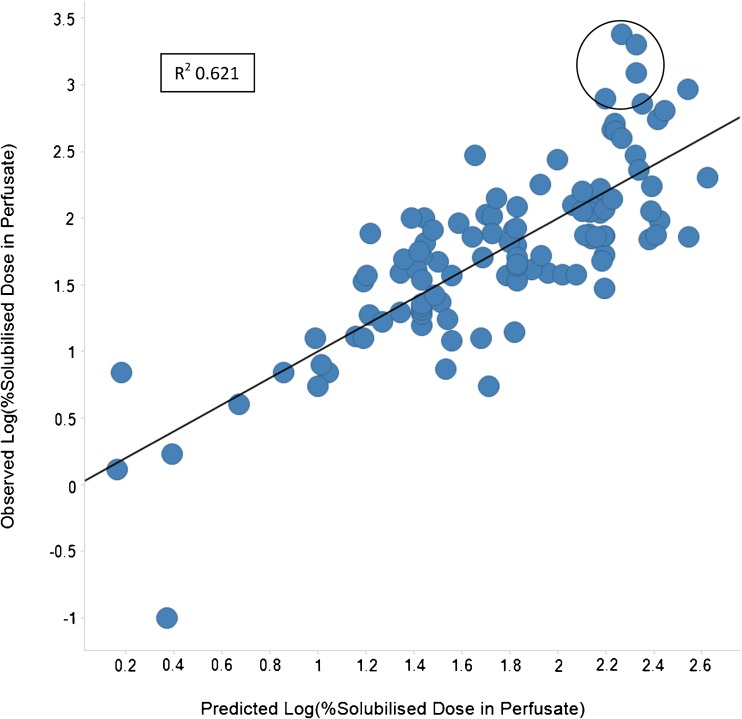
The output from the OPLS model showing the correlation between predicted Log%SDiP on the x axis and observed Log%SDiP on the y axis. The model output R^2^ and Q^2^ values were 0.621 and 0.491 respectively. Circled area highlights potential for model to under-predict for some “high” classification compounds (>300%SDiP).

Output from the OPLS model showing the correlation between predicted and observed Log%SDiP is shown in Fig. 5; the compounds circled suggest that the model under-predicts the actual value for observations of approximately >300%SDiP (~2.5 on the log scale), but correctly assigns them as being in the high (>100%) category.

### Validation of the QSAR model with a “Test set” of 9 compounds

Having built a computational model which can predict pulmonary absorption in the IPRLu model for inhaled compounds based on calculated physicochemical descriptors we then evaluated the performance of the model using an additional 9 test compounds which had not been included in the Training set.

**Fig. 6:**
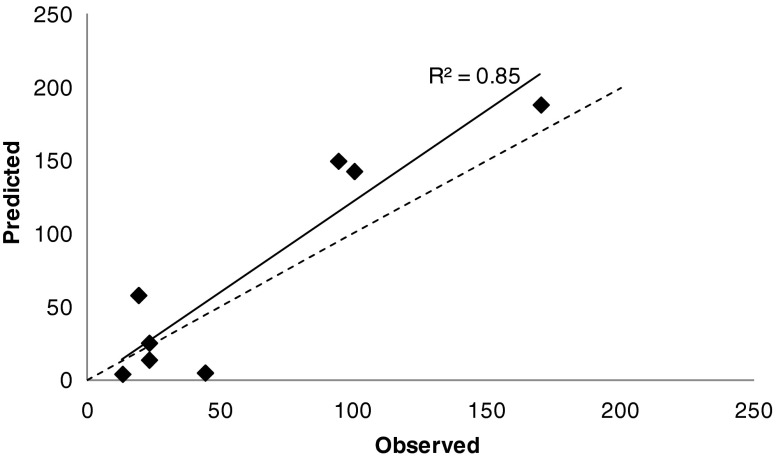
Comparison between predicted and mean observed %SDiP for the “Test set”. *Dashed line* = unity. *Solid* = linear trendline with R^2^ of 0.85 with outlier removed.

The observed IPRLu data with the 9 “Test set” compounds are compared to the QSAR model predictions in Table V. Overall the QSAR model performed well, especially when predicting the classification of each compound. 67% (6/9) of the “Test set” were categorised correctly based on a comparison of the predicted %SDiP with the observed mean data. This increased to 89% (8/9) when comparing to the observed range rather than the mean. Although an absolute %SDiP value could not be determined for GSK_A due to the limit of detection of the HPLC-MS/MS assay, <13%SDiP indicates negligible pulmonary absorption, supported by complete recovery of the dose from the lungs at the end of the experiment, this is in keeping with the prediction that GSK_A would be in the “Low” category. For the remaining “Test set” compounds mean observed values of %SDiP were predicted within 2-fold of the observed mean for 63% (5/8) of the compounds. A comparison of the observed *versus* predicted %SDiP for the “Test set” compounds (Fig. 6) displayed an R^2^ of 0.85. GSK_I was excluded from this analysis because, although categorised correctly, its observed value of >350%SDiP falls within the circled area in Fig. 5 and therefore places this compound in a region where the model is known to under predict the absolute value.

**Table V:** Comparison of Observed and Predicted %SDiP Data for the 9 “Test Set” Compounds

Compound	Observed mean % [range] (category)	Predicted % (category)	Accuracy of prediction
GSK_A	<13 (low/mod)	4 (low)	Good Prediction
GSK_B	170 [169–170] (high)	188 (high)	Good Prediction
GSK_C	19 [15–23] (mod)	58 (mod)	Same category but >2fold
GSK_D	23 [15–30] (mod)	26 (mod)	Good Prediction
GSK_E	44 [32–56] (mod)	5 (low)	Underestimate
GSK_F	23 [16–29] (mod)	14 (mod)	Good Prediction
GSK_G	94 [77–111] (mod)	150 (high)	Prediction within 2 fold
GSK_H	100 [90–111] (mod)	143 (high)	Prediction within 2 fold
GSK_I	352 [309–395] (high)	122 (high)	Same category but >2fold

Observed data were generated by the IPRLu model and mean values are displayed along with the range (n = 2), predicted values are the anti-Log of the output from the OPLS model

The values of %SDiP were classified as Low (<10%) *i.e.* compounds which did not cross into the perfusate to any great extent, Moderate (10–100%) and High (>100%) indicting that absorption is not limited by solubility in the dose formulation

Overall, based on the outcome of the model validation with the compound “Test set” our data successfully validated the QSAR model for prospective use.

## Discussion

One of the main challenges in inhaled drug discovery is that, unlike oral drug discovery, many of the physical properties governing the lung disposition of small molecule inhaled drugs have not been clearly defined. Much of the information in the literature concerning the inhaled route of administration focuses on particulates, macromolecules or very water soluble molecules. Consequently, characterization and optimization of molecules for inhaled delivery rely largely on empirical testing in *ex vivo* and/or *in vivo* pre-clinical models. Here we present a novel in silico model constructed using the largest, most diverse and relevant data set available to date, combining both marketed inhaled drugs and novel inhaled compounds with less established mechanisms of action. We subsequently evaluated our in silico model using a compound “Test set”, which generated robust prospective predictions, confirming the applicability of our IPRLu model in ranking compounds according to their lung disposition.

In our current study, we used a dataset of 98 compounds that were tested in an IPRLu model to build a QSAR model in order to predict the %SDiP following intratracheal administration as an aqueous solution/suspension. Tronde (24) describes an alternate QSAR model, however this was based on a smaller IPRLu model dataset of 10 compounds, 3 of which were inhaled marketed drugs. We have sought to extend this approach with a dataset of over 100 compounds across our “Training set” and “Test set”. As our dataset included marketed inhaled drugs and novel inhaled drug discovery compounds, our novel QSAR model enables drug discovery programme teams to compare QSAR model predictions for new chemical entities with similar data generated for marketed inhaled compounds.

The marketed compound set can be placed into 2 distinct groups based on the IPRLu model data and their physicochemical descriptors.

For the first group of compounds (%SDiP ≅ %TDiP) displaying a hydrophilic mean LogP of -0.3 and where the dose is mainly in solution rather than a suspension, poor permeability and potentially tissue binding are likely factors limiting passage across the IPRLu model rather than poor solubility. This group includes indacaterol, ambroxol, formoterol fumerate, ipratropium bromide, amiloride, lidocaine and zanamivir. All display moderate %SDiP values ranging from 12% for indacaterol (with a lung absorption half-life of 273 minutes) to 100% for zanamivir (with a lung absorption half-life of 7 minutes). These data show how readily even zwitterionic compounds like zanamivir with low permeability can be absorbed across the lung epithelium, when they would not be absorbed across the GI tract (35).

The second group of compounds (%SDiP > %TDiP) display a lipophilic mean LogP of 3.6 and include: flunisolide, montelukast, fluticasone propionate, fluticasone furoate, nedocromil, tacrolimus, budesonide, salmeterol and salbutamol. For this group, which were dosed as suspensions passage across the lung is likely to be mainly limited by solubility and slow dissolution rate in the dose vehicle or lung lining fluid. For instance, flunisolide and montelukast display 100% SDiP suggesting that no further dissolution of the drug occurs once instilled in the lung but that the solubilised drug in the dose formulation is permeable and crosses into the perfusate. However, fluticasone propionate, fluticasone furoate, nedocromil, tacrolimus and budesonide display >100%SDiP indicating some dissolution of particulate drug during the IPRLu model perfusion experiment and good permeability, but with solubility limiting %TDiP to <100%. Finally for compounds displaying <100% SDiP, tissue binding and permeability may also contribute to limiting absorption across the IPRLu model.

In this paper our observations show that the factors driving pulmonary absorption are diverse and that this diversity is well reflected in inhaled marketed compounds which were used to construct our QSAR model.

Solubility is an important driving factor of pulmonary absorption. However differences in solubility between salt forms and different physical forms of the same parent compound cannot be predicted from parent compound structure. The IPRLu model data in Fig. 1. showed that the solubility of the salt form in the dose vehicle correlated with the %TDiP but resulted in similar %SDiP. This finding confirmed that solubility in the dose vehicle and consequently whether the dose is administered as an aqueous solution or suspension, is an important factor determining the profile of compounds in the IPRLu model and underlines the importance of determining experimentally the % dose in solution. This finding also supports the building of a QSAR model on %SDiP rather than %TDiP, because the descriptors used to build the model are calculated from the parent molecular structure and therefore would be unable to predict differences arising from changes in salt form, physical form or particle size. This approach removes the inevitable sources of variability associated with solubility of different batches, salt forms and dose vehicles.

Our QSAR model is built on %SDiP and hence predicts the inherent ability of the parent compound to cross the lungs and appear in the perfusate once it is in solution.

The model also flags compounds (classified as Low) which are more likely to accumulate in the lung upon repeat dosing and therefore pose an increased risk of developability issues. This is in keeping with IPRLu model data obtained with the marketed compounds, as none of these display low absorption (classified as <10% of the solubilised or total dose in the perfusate at 20 minutes).

In attempting to maximise the number of compounds in any ADME dataset, there is an increased risk of introducing noise and this IPRLu model dataset is no exception. Driven by the speed and cost constraints of a drug discovery setting, the majority of the data used to build this model was carried out in duplicate and often administered as a cassette of up to 4 compounds to reduce the number of animals used. Data in Table I shows however that the majority of compounds display duplicate % dose in perfusate values that are within 2 fold, suggesting limited variability for most compounds. One of the advantages of selecting an OPLS model is to separate out the additional sources of variability and focus the variance attributed to the y data into principal component 1. As a result an acceptable model was built (R^2^ 0.621, Q^2^ 0.491) which proved to be robust against the “Test set”.

As shown in Table III and Fig. 4, calculated descriptors that positively correlate with Log%SDiP are mainly associated with permeability and hydrophobicity whereas descriptors that negatively correlate are mainly associated with charge, ionisation and size. These observations are in keeping with those reported by Tronde, *i.e.* rate of absorption across the IPRLu model positively correlating with hydrophobicity and negatively correlating with %PSA (24). They are also in keeping with earlier *in vivo* work in the rat reporting absorption from the lung to correlate inversely with molecular weight (15;16).

Log%SDiP also correlates with the predicted ADME endpoints of a number of inhouse QSAR models. For example FA_rat_v1.logFA_score which predicts fraction absorbed following oral administration in the rat and is also driven by permeability and solubility. In addition Pgp_v31.Pgp_Score predicts the likelihood of a compound being a substrate for the ABC active efflux transporter P-glycoprotein. This does not necessarily suggest that by being a substrate for Pgp the compound is more likely to be transported across the lung into the perfusate, just that similar calculated descriptors underpin both the IPRLu QSAR model and the Pgp QSAR model. In this case hydrophobicity correlates with both models in keeping with the general idea that Pgp acts as a transporter for mainly hydrophobic substrates (36). As an endpoint %SDiP also has the potential to differentiate between acidic, basic and neutral compounds due to their different distribution characteristics. For example in the IPRLu QSAR model “neutral ionised form” is a positive coefficient contributing to an increase in %SDiP whereas “basic ionised form” is a negative coefficient contributing to a reduced %SDiP. This may reflect the potential for basic molecules to distribute into lysosomal vesicles and bind more readily to acidic phospholipids. When considering the endpoint %TDiP, the correlation was reversed with “basic” and “neutral ionised form” emerging as positive and negative coefficients respectively. This observation is in keeping with solubility driving %TDiP as basic compounds are generally more soluble than neutral compounds. Interestingly this differentiation between basic and neutral compounds was not evident when considering lung absorption half-life as the endpoint. “Acidic ionised form” did not emerge as a significant coefficient in the QSAR model, possibly because there were only 8 acidic compounds in the “Training set”. The %SDiP also displayed trends with *in vitro* physicochemical measurements such as hydrophobicity, permeability and protein binding which were in keeping with the calculated descriptors. However, the QSAR model was built entirely from in silico inputs to enable its application during compound design.

The QSAR model described here offers benefits for application in a drug discovery setting, particularly in reducing the routine need for animal studies. The model can be used as part of a strategy to reduce systemic exposure and increase duration of action at the target site for respiratory indications. Although not as appropriate for predicting clinical profiles as the model described by Jones and Harrison (37), the QSAR model is a valuable ranking tool which can direct inhaled drug design, whilst the IPRLu model can be reserved for more mechanistic type studies.

Topical delivery for respiratory diseases has the advantage of delivering therapeutic levels of compound to the target organ from a relatively low dose. Optimisation of inhaled medicines often focuses on improving the therapeutic index by limiting systemic exposure to avoid toxicity, or by increasing the duration of action of compounds at the site of action. Both of these can in principle be achieved by slowing the passage of drug across the lung barrier, and therefore retention of compound in its soluble form at the target site is generally considered to be a desirable property. The ultimate aim is to increase the duration that compound is free to engage at the target site. Examples include the glucocorticoid fluticasone furoate which has shown high retention and activity in nasal tissue *ex vivo* (38) and the muscarinic antagonist tiotropium bromide for which a slow receptor off-rate is proposed to provide a long duration of action (39).

It is of course difficult to compare the IPRLu model data on the marketed compounds in Table I with any clinical data because the latter is often generated following a low dose, typically <1 mg administered as an inhaled dry powder. Clinical plasma profiles, which are often not available following such low doses, are dependent upon salt form, particle size, device and plasma clearance, none of which feature in this IPRLu model. For these reasons it is also unclear how to scale the IPRLu model data to *in vivo*. However, consistencies reported between the IPRLu model and *in vivo* models are encouraging (24;25) and suggest that the IPRLu model and the resulting QSAR model described here are useful ranking tools. The advantage of using a QSAR model is that predictions can be made very quickly on molecular structures prior to synthesis. This reduces the number of animal studies by selecting compounds with appropriate or diverse properties based on the QSAR model predictions, enabling more rapid investigation of the relationship between lung retention and efficacy or toxicity *in vivo*.

The generation of data using the IPRLu model can then be reserved for more mechanistic studies for example investigating links between different salt forms and dose vehicles with efficacy in PD models, investigating the impact of active transporters on the pulmonary disposition of drug substrates (40–42), or in combination with PD endpoints from the same model (43,44).

## Conclusion

The novel QSAR model described here can replace routine generation of IPRLu model data for ranking and classifying compounds prior to synthesis. It will also provide scientists working in the field of inhaled drug discovery with a deeper understanding of the physicochemical drivers of pulmonary absorption. These QSAR based predictions can help prioritise compounds and aid in the interpretation of efficacy, lung accumulation or systemic toxicity endpoints across inhaled drug discovery and development programmes.

## ABBREVIATIONS

ADME: Absorption, distribution, metabolism and excretion
ADMET: Absorption, distribution, metabolism, excretion and toxicology
BSA: Bovine serum albumin
CD: Caesarian derived
FB: Free base
GSK: GlaxoSmithKline
HCl: Hydrochloride salt
HNA: Hydroxynapthoate
HPLC: High pressure liquid chromatography
HPLC-MS/MS: High pressure liquid chromatography tandem mass spectrometry
IPRLu: Isolated perfused respiring rat lung
MDCK: Madine Darby canine kidney
OPLS: Orthogonal partial least squares
QSAR: Quantitative structure-activity relationship
SDiP: Solubilised dose in perfusate at 20mins
TDiP: Total dose in perfusate at 20mins

## References

[1] Lipinski CA, Lombardo F, Dominy BW, Feeney PJ (1997). Experimental and computational approaches to estimate solubility and permeability in drug discovery and development settings. Adv Drug Deliv Rev.

[2] Gleeson MP (2008). Generation of a set of simple, interpretable ADMET rules of thumb. J Med Chem.

[3] Ritchie TJ, Macdonald SJ (2009). The impact of aromatic ring count on compound developability--are too many aromatic rings a liability in drug design?. Drug Discov Today.

[4] Young RJ, Green DV, Luscombe CN, Hill AP (2011). Getting physical in drug discovery II: the impact of chromatographic hydrophobicity measurements and aromaticity. Drug Discov Today.

[5] Hughes JD, Blagg J, Price DA, Bailey S, DeCrescenzo GA, Devraj RV (2008). Physiochemical drug properties associated with in vivo toxicological outcomes. Bioorg Med Chem Lett.

[6] Leeson PD, Springthorpe B (2007). The influence of drug-like concepts on decision-making in medicinal chemistry. Nat Rev Drug Discov.

[7] Price DA, Blagg J, Jones L, Greene N, Wager T (2009). Physicochemical drug properties associated with in vivo toxicological outcomes: a review. Expert Opin Drug Metab Toxicol.

[8] Gleeson MP, Hersey A, Montanari D, Overington J (2011). Probing the links between in vitro potency, ADMET and physicochemical parameters. Nat Rev Drug Discov.

[9] Matero S, Lahtela-Kakkonen M, Korhonen O, Ketolainen J, Lappalainen R, Poso A (2006). Chemical space of orally active compounds. Chemom Intell Lab Syst.

[10] Varma MV, Obach RS, Rotter C, Miller HR, Chang G, Steyn SJ (2010). Physicochemical space for optimum oral bioavailability: contribution of human intestinal absorption and first-pass elimination. J Med Chem.

[11] Veber DF, Johnson SR, Cheng HY, Smith BR, Ward KW, Kopple KD (2002). Molecular properties that influence the oral bioavailability of drug candidates. J Med Chem.

[12] Byron PR, Patton JS (1994). Drug delivery via the respiratory tract. J Aerosol Med.

[13] Patton JS, Byron PR (2007). Inhaling medicines: delivering drugs to the body through the lungs. Nat Rev Drug Discov.

[14] Chan TL, Lee PS, Hering WE. Deposition and clearance of inhaled diesel exhaust particles in the respiratory tract of Fischer rats. 1981;1(2):77–82. 10.1002/jat.2550010206 6206117

[15] Patton JS (1996). Mechanisms of macromolecule absorption by the lungs. Adv Drug Deliv Rev.

[16] Enna SJ, Schanker LS (1972). Absorption of saccharides and urea from the rat lung. Am J Physiol.

[17] Henderson RF, Bechtold WE, Medinsky MA, Fischer JP, Lee TT (1988). The effect of molecular weight/lipophilicity on clearance of organic compounds from lungs. Toxicol Appl Pharmacol.

[18] Ritchie TJ, Luscombe CN, Macdonald SJ (2009). Analysis of the calculated physicochemical properties of respiratory drugs: can we design for inhaled drugs yet?. J Chem Inf Model.

[19] Byron PR, Niven RW (1988). A novel dosing method for drug administration to the airways of the isolated perfused rat lung. J Pharm Sci.

[20] Jeppsson AB, Nilsson E, Waldeck B (1994). Formoterol and salmeterol are both long acting compared to terbutaline in the isolated perfused and ventilated guinea-pig lung. Eur J Pharmacol.

[21] Mehendale HM, Angevine LS, Ohmiya Y (1981). The isolated perfused lung—a critical evaluation. Toxicology.

[22] Ryrfeldt A, Persson G, Nilsson E (1989). Pulmonary disposition of the potent glucocorticoid budesonide, evaluated in an isolated perfused rat lung model. Biochem Pharmacol.

[23] Tronde A, Krondahl E, von Euler-Chelpin H, Brunmark P, Bengtsson UH, Ekstrom G (2002). High airway-to-blood transport of an opioid tetrapeptide in the isolated rat lung after aerosol delivery. Peptides.

[24] Tronde A, Norden B, Jeppsson AB, Brunmark P, Nilsson E, Lennernas H (2003). Drug absorption from the isolated perfused rat lung—correlations with drug physicochemical properties and epithelial permeability. J Drug Target.

[25] Selg E, Ewing P, Acevedo F, Sjoberg CO, Ryrfeldt A, Gerde P (2013). Dry powder inhalation exposures of the endotracheally intubated rat lung, ex vivo and in vivo: the pulmonary pharmacokinetics of fluticasone furoate. J Aerosol Med Pulm Drug Deliv.

[26] Brain JD, Knudson DE, Sorokin SP, Davis MA (1976). Pulmonary distribution of particles given by intratracheal instillation or by aerosol inhalation. Environ Res.

[27] Fisher AB, Dodia C, Linask J (1980). Perfusate composition and edema formation in isolated rat lungs. Exp Lung Res.

[28] Geladi P, Kowalski BR (1986). Partial least-squares regression: a tutorial. Anal Chim Acta.

[29] Wold S, Geladi P, Esbensen K, Öhman J (1987). Multi-way principal components-and PLS-analysis. J Chemom.

[30] Platts JA, Abraham MH, Butina D, Hersey A (2000). Estimation of molecular linear free energy relationship descriptors by a group contribution approach. 2. prediction of partition coefficients. J Chem Inf Model.

[31] Cho MJ, Thompson DP, Cramer CT, Vidmar TJ, Scieszka JF (1989). The Madin Darby canine kidney (MDCK) epithelial cell monolayer as a model cellular transport barrier. Pharm Res.

[32] Valko K, Bevan C, Reynolds D (1997). Chromatographic hydrophobicity index by fast-gradient RP-HPLC: a high-throughput alternative to log P/log D. Anal Chem.

[33] Andrews PR, Craik DJ, Martin JL (1984). Functional group contributions to drug-receptor interactions. J Med Chem.

[34] Ertl P, Rohde B, Selzer P (2000). Fast calculation of molecular polar surface area as a sum of fragment-based contributions and its application to the prediction of drug transport properties. J Med Chem.

[35] Gupta SV, Gupta D, Sun J, Dahan A, Tsume Y, Hilfinger J (2011). Enhancing the intestinal membrane permeability of zanamivir: a carrier mediated prodrug approach. Mol Pharm.

[36] Schinkel AH, Jonker JW (2003). Mammalian drug efflux transporters of the ATP binding cassette (ABC) family: an overview. Adv Drug Deliv Rev.

[37] Jones RM, Harrison A (2012). A new methodology for predicting human pharmacokinetics for inhaled drugs from oratracheal pharmacokinetic data in rats. Xenobiotica.

[38] Baumann D, Bachert C, Hogger P (2009). Dissolution in nasal fluid, retention and anti-inflammatory activity of fluticasone furoate in human nasal tissue ex vivo. Clin Exp Allergy.

[39] Casarosa P, Bouyssou T, Germeyer S, Schnapp A, Gantner F, Pieper M (2009). Preclinical evaluation of long-acting muscarinic antagonists: comparison of tiotropium and investigational drugs. J Pharmacol Exp Ther.

[40] Al-Jayyoussi G, Price DF, Francombe D, Taylor G, Smith MW, Morris C (2013). Selectivity in the impact of P-glycoprotein upon pulmonary absorption of airway-dosed substrates: a study in ex vivo lung models using chemical inhibition and genetic knockout. J Pharm Sci.

[41] Bosquillon C (2010). Drug transporters in the lung—do they play a role in the biopharmaceutics of inhaled drugs?. J Pharm Sci.

[42] Gumbleton M, Al-Jayyoussi G, Crandon-Lewis A, Francombe D, Kreitmeyr K, Morris CJ (2011). Spatial expression and functionality of drug transporters in the intact lung. Object Further Res.

[43] Gnadt M, Trammer B, Kardziev B, Bayliss MK, Edwards CD, Schmidt M (2012). Comparison of the bronchodilating effects of inhaled beta(2)-agonists after methacholine challenge in a human lung reperfusion model. Eur J Pharm Biopharm.

[44] Gnadt M, Trammer B, Freiwald M, Kardziev B, Bayliss MK, Edwards CD (2012). Methacholine delays pulmonary absorption of inhaled beta(2)-agonists due to competition for organic cation/carnitine transporters. Pulm Pharmacol Ther.

